# Leisure activities, self-reported health, anxiety, and depression in Chinese older adults: a structural equation model analysis

**DOI:** 10.3389/fpubh.2025.1459236

**Published:** 2025-05-09

**Authors:** Keying Song, Yawen Chang, Zijian Zhao, Karuppasamy Govindasamy

**Affiliations:** ^1^Gdansk University of Physical Education and Sport, Gdańsk, Poland; ^2^Department of Physical Education, Zhengzhou University, Zhengzhou, China; ^3^Department of Sports, Recreation and Wellness, Symbiosis International (Deemed University), Hyderabad Campus, Modallaguda (V), Nandigama (M), Rangareddy, Telangana, India

**Keywords:** leisure activities, self-reported health, anxiety, depression, Chinese older adults

## Abstract

**Objectives:**

This study aimed to explore the interrelationships among leisure activities, self-reported health (SRH), anxiety, and depression in Chinese older adults using structural equation modeling (SEM).

**Methods:**

Data from the 2018 Chinese Longitudinal Healthy Longevity Survey (CLHLS) were analyzed and 9,859 older adults were included in the analysis. The SEM was employed to examine the direct and indirect effects of leisure activities on depression through SRH and anxiety.

**Results:**

Leisure activities significantly positively influenced SRH (*β* = 0.18, *p* < 0.001) and negatively affected depression (*β* = −0.16, *p* < 0.001) and anxiety (*β* = −0.09, *p* < 0.001). SRH decreased depression (*β* = −0.33, *p* < 0.001) and anxiety (*β* = −0.33, *p* < 0.001), while anxiety increased depression (*β* = 0.49, *p* < 0.001). SRH and anxiety mediated the relationship between leisure activities and depression.

**Conclusion:**

The findings support the proposed model and suggest that promoting leisure activities can improve mental health in Chinese older adults.

## 1 Introduction

Mental health is a growing issue worldwide. According to World Health Organization (WHO), nearly 1 billion people worldwide suffer from mental disorders, equivalent to about 13% of the global population (1). Depressive and anxiety disorders are the leading contributors to this burden. Depression affects an estimated 280 million people globally, while anxiety disorders affect around 300 million people (2). Furthermore, with the outbreak of COVID-19 pandemic, a 25% increase in the prevalence of anxiety and depression worldwide has been triggered (3). Depressive disorders, peaking in older adulthood, affect approximately 63 million people or 7% of the population aged 60 years and older worldwide (4). As China has entered an aging society, the proportion of people aged 60 and over in China reached 18.70%, and the proportion of people aged 65 and over reached 13.50%, with a trend of increasing year by year (5). Improving mental health, particularly preventing anxiety and depression among older adults, has become a serious public health issue in China, especially after the COVID-19 epidemic.

Leisure activities have been identified as a positive method for improving health and well-being among older adults. These activities, which include physical, cognitive, intellectual, and social engagements, are associated with better health outcomes, successful aging and overall well-being in older adults (6–9). The participation and exploration of leisure activities among older adults have been significantly linked to depression levels and quality of life (10). Successful engagement in leisure activities during old age can help alleviate psychological stress, enhance life satisfaction, and improve overall quality of life (11). Additionally, these activities have been shown to be associated with reduced all-cause mortality among older adults (12, 13).

Although many studies have examined factors affecting depression among older adults, none have simultaneously examined the relationships among leisure activities, SRH, anxiety, and depression. In addition, there have been no studies to investigate the relationships between leisure activities and depression through mediating factors such as SRH or anxiety. Therefore, this study proposes and tests a conceptual model explaining these interrelationships using CLHLS data.

### 1.1 Relationships among leisure activities, SRH, anxiety, and depression

Numerous studies have highlighted the positive impact of leisure activities on the health of older adults. For instance, Yang et al. (7) demonstrated that consistent engagement in productive physical activity can enhance perceived health-related quality of life among older individuals. Similarly, Sala et al. (8) found that active participation in leisure activities helps maintain cognitive, physical, and mental health in this population. Other researchers, such as Guerrero et al. (14) have shown that leisure activities can prevent dementia and other diseases in older adults. Additionally, studies by Lin et al. (15) have identified positive associations between physical activity and SRH.

Leisure activities have also been found to prevent depression and anxiety in older adults (16, 17) and are considered a beneficial method for improving mental health. Specifically, several studies have reported that physical activity can reduce symptoms of depression and anxiety (18, 19). For example, Wanjau et al. (20) found that higher levels of physical activity are inversely related to incident depression and anxiety, suggesting that inactivity may be causally linked to these conditions. Kim et al. (21) also observed that participation in leisure physical activity and resistance exercise is associated with a lower prevalence of depressive symptoms among Korean adults. Mammen et al. (22) identified a significant inverse relationship between baseline physical activity and depression in a review of 30 primary studies. McDowell et al. (23) conducted a systematic review and meta-analysis of prospective cohort studies, finding that physical activity is associated with lower odds of self-reported anxiety symptoms, diagnosis of any anxiety disorder, and diagnosis of generalized anxiety disorder. Schuch et al. (24) found that high self-reported physical activity levels are associated with lower rates of incident anxiety compared to low physical activity levels. As a result, physical activity has been proposed as an adjunct treatment for depression and anxiety (25).

SRH is a subjective measure that encompasses biological, mental, social, and functional aspects of a person’s well-being, including individual and cultural beliefs and health behaviors (26). Research indicates that SRH effectively predicts mortality (27) and SRH is a significant predictor of current health status (28), an important indicator of depression (29), and is associated with quality of life and depression (30). For example, Badawi et al. (31) found that fair or poor self-rated health at baseline predicts a twofold increased risk for major depression at follow-up, even after adjusting for socio-demographic characteristics, lifestyle behaviors, disability, and diabetes. Depression has also been linked to poor self-rated health and increased mortality in the general population (32). More recently, Shen and Zou (33) identified an inverse nonlinear association between cardiovascular health and depression.

Studies have also shown that individuals with poorer SRH status often exhibit higher levels of anxiety symptoms. This association may stem from the negative impact of mental health problems on an individual’s overall perception of health (34). Additionally, the frequent occurrence of anxiety symptoms is closely related to a decline in health-related quality of life, and anxiety may reduce an individual’s overall satisfaction with their health by affecting their emotional state and daily functioning (35), indicating that anxiety affects not only mental health but also an individual’s subjective evaluation of their own health through psychological mechanisms (36).

In addition, studies have also shown SRH may play a mediating role between leisure activities and depression and actively engaging in leisure activities can enhance an individual’s SRH, which in turn reduces depressive symptoms (37, 38).

With regards to the relationship between anxiety and depression, comorbid depression and anxiety are highly prevalent conditions (39). Study (40) emphasized that the overlap between depression and anxiety symptoms is central to classifying affective disorders, as most patients with depression also have anxiety, and vice versa. Evidence also suggested genetic and neurobiologic similarities between depressive and anxiety disorders (41). In relation to the evolution of their comorbidity, studies demonstrated that anxiety disorders generally precede the presentation of major depressive disorder (42). A meta-analysis (43) found that anxiety and depression are bidirectional risk factors, and treating one disorder can significantly reduce the symptoms of the other. Another study (44) indicated that treatments targeting anxiety (particularly CBT and medication) can significantly improve the prognosis of patients with comorbid depression.

### 1.2 Research hypotheses

Drawing on the existing literature, we formulate the following hypotheses:

H1: Leisure activities will have a positive and direct impact on SRH among older adults in China.

H2: Leisure activities will have a negative effect on depression among older adults in China.

H3: Leisure activities will have a negative effect on anxiety among older adults in China.

H4: SRH will have a negative influence on depression among older adults in China.

H5: SRH will have a negative influence on anxiety among older adults in China.

H6: Anxiety will have a positive effect on depression among older adults in China.

**Figure 1:**
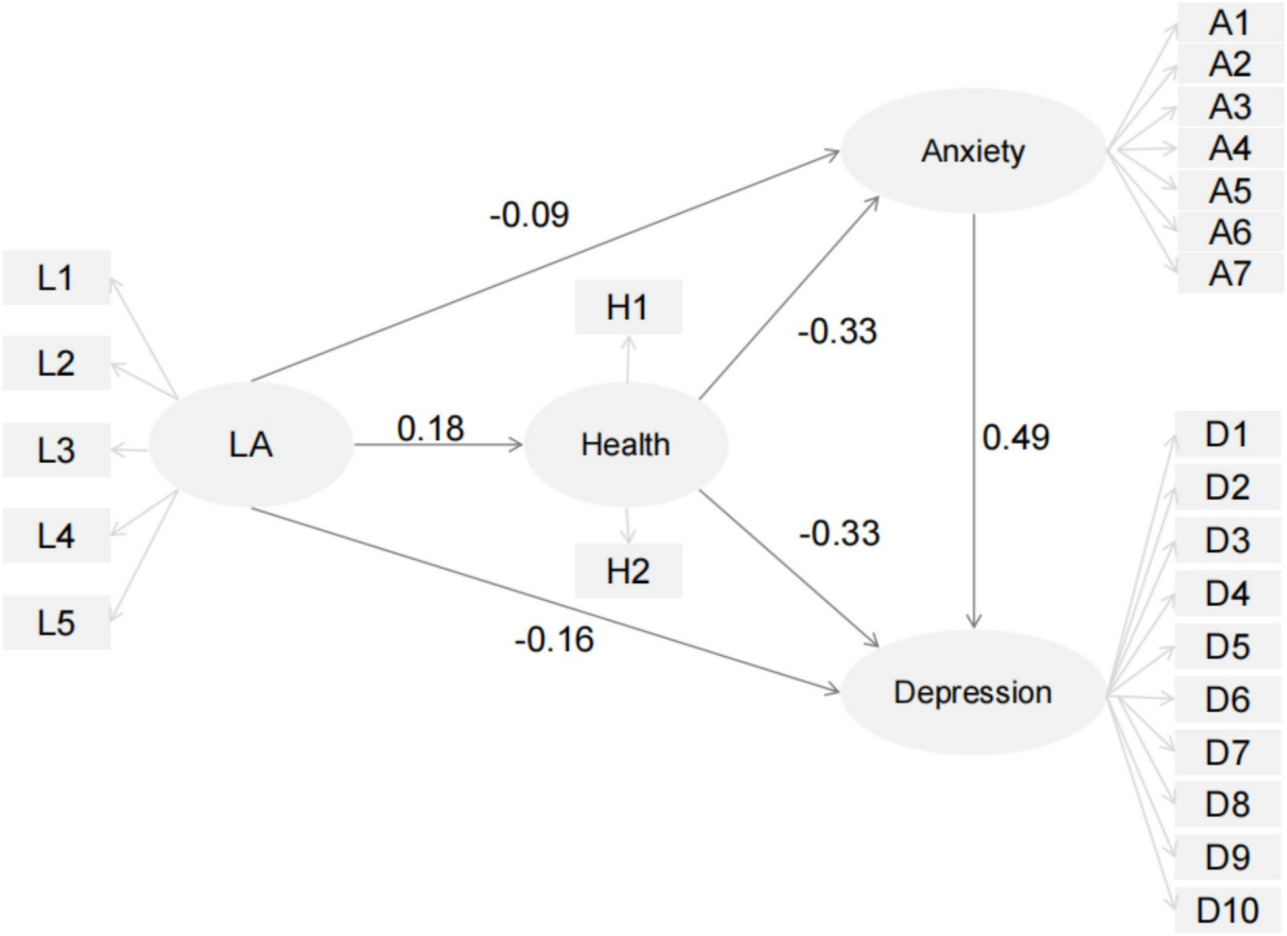
Proposed model of the influence of leisure activities, self-reported health, and anxiety on depression in older adults and structural test for the relationships among those variables. Of LA, leisure activities.

H7: SRH and anxiety will mediate the relationship between leisure activities and depression among older adults in China (Figure 1)

## 2 Materials and methods

### 2.1 Data source and samples

The present study utilized data from the 2018 wave of the CLHLS. It covers 23 out of 31 provinces in China and includes 15,874 respondents aged 50 years and older. The survey comprises more than 750 questions, covering extensive data on demographics, socioeconomic conditions, psychological traits, social participation, and health conditions. All data were collected via face-to-face interviews during in-home visits (45). The study was approved by the Ethics Committee of Peking University (IRB00001052–13074) (46). For more detailed information regarding the CLHLS, including the sampling design, response rates, attrition, and systematic assessments of data quality, readers are referred to previous publications (47, 48). Our research is a secondary analysis using data from the CLHLS. All procedures performed in studies involving human participants were in accordance with the ethical standards of the Ethics Committee of Peking University and with the 1964 Helsinki declaration and its later amendments or comparable ethical standards.

**Figure 2:**
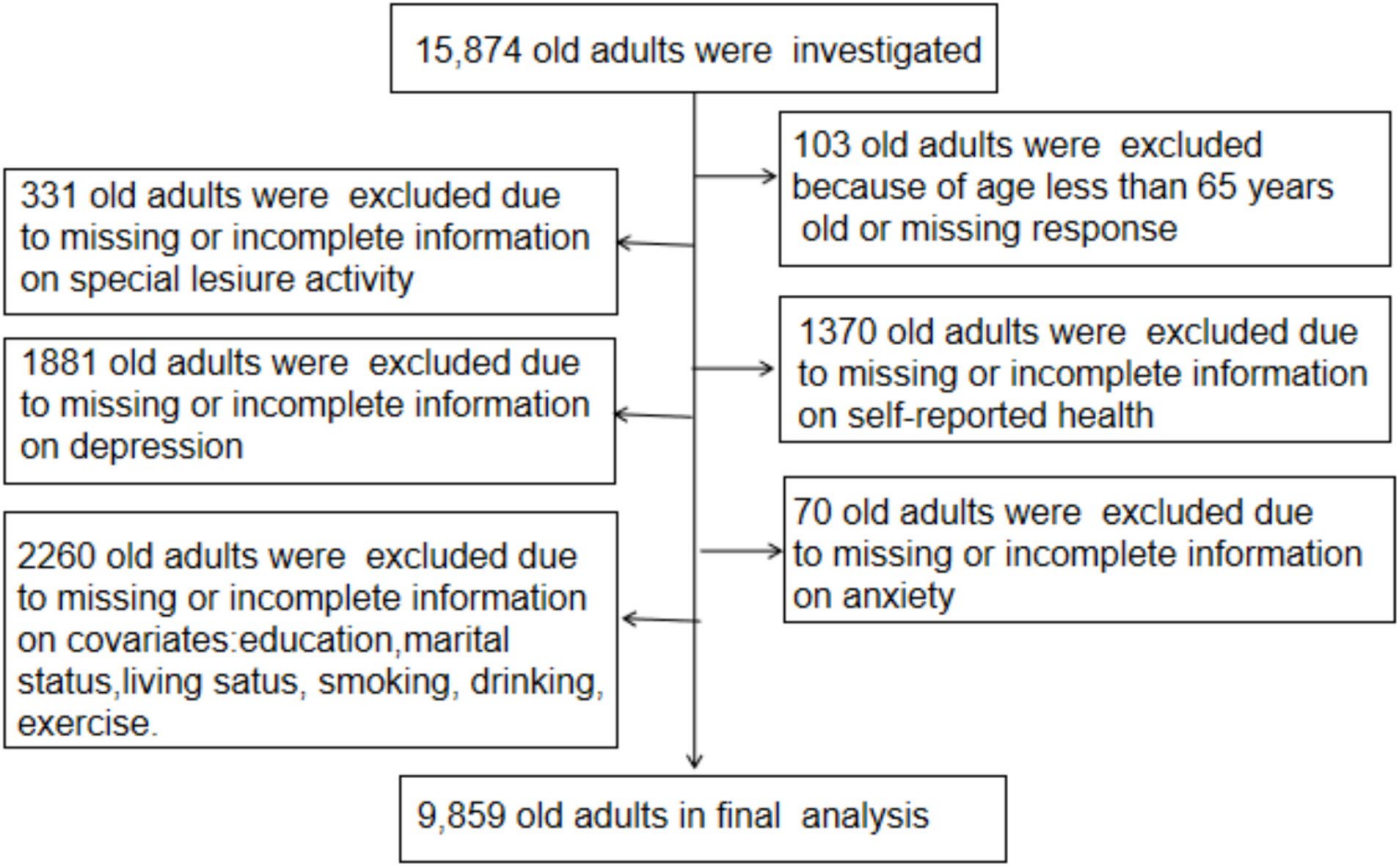
Flowchart of participants included in this study.

For the current analysis, participants were included if they met the following criteria: (a) aged 65 years and older; (b) complete information on variables related to leisure activities, SRH, anxiety, and depression; and (c) complete data on variables such as age, sex, residence, education level, marital status, living status (co-residence), smoking, drinking, and exercise. A total of 15,874 participants were surveyed in the 2018 wave of the CLHLS. After excluding individuals who did not meet the inclusion criteria, the final analysis included 9,859 older adults (Figure 2).

### 2.2 Assessment of leisure activities

A five-item index was used to assess participants’ engagement in leisure activities. Participants were asked, “Do you participate in the following activities?” The activities included: (a) gardening, (b) reading newspapers or books, (c) playing cards or mahjong, (d) watching TV or listening to the radio, and (e) participating in social activities. Responses for each activity were recorded on a five-point frequency scale: 1 = almost every day, 2 = not daily but at least once a week, 3 = not weekly but at least once a month, 4 = not monthly but sometimes, and 5 = never. To ensure that higher scores indicated greater participation in leisure activities, the scores were reversed such that a higher score corresponded to a higher level engagement.

### 2.3 Assessment of self-reported health

SRH was assessed using two questions: (a) “How do you rate your health at present?” with response options ranging from 1 (very good) to 5 (very bad); and (b) “How do you rate your health at present compared with 1 year ago?” with response options ranging from 1 (much better) to 5 (much worse). To ensure that higher scores indicated better health, the scores were reversed such that a higher score corresponded to better health status.

### 2.4 Assessment of anxiety

Anxiety symptoms were assessed using the 7-item Generalized Anxiety Disorder scale (GAD-7) (49), which has been validated among Chinese old adults (50, 51). Participants were asked, “In the last 2 weeks, how often have you experienced the following symptoms?” Seven items were included. Each item was rated on a 4-point scale: 0 = not at all, 1 = several days, 2 = more than half the days, and 3 = nearly every day. The total score ranges from 0 to 21, with higher scores indicating more severe anxiety symptoms. Previous research has confirmed the reliability and validity of the GAD-7 in measuring anxiety symptoms (52). In the current study, the Cronbach’s alpha coefficient for the anxiety scale was 0.92, indicating high internal consistency.

### 2.5 Assessment of depression

Depression was measured using the 10-item Center for Epidemiological Studies Depression Scale (CESD-10), which has demonstrated adequate reliability (53). Participants in the 2018 CLHLS were asked to rate “how often you felt this way during the past week” for each item. Responses were recorded on a five-point scale: 1 = always, 2 = often, 3 = sometimes, 4 = seldom, and 5 = never. To ensure that higher scores indicated more severe depression, the scores were reversed such that a higher score corresponded to greater depression severity. The Cronbach’s alpha coefficient for the CESD-10 in this study was 0.81, indicating good internal consistency.

### 2.6 Assessment of other variables

Several factors related to demographics, socioeconomic status, and health were included in this study, as they may be potential risk factors for depression and anxiety in older adults. These factors included age, sex, residence, education level, marital status, living status (co-residence), smoking, drinking, and exercise. Residence was classified as “urban” or “rural.” Education level was assessed by asking participants the number of years they had attended school. Marital status was categorized as “married and living with spouse,” “widowed, “or “others (married but not living with spouse, divorced, or never married).” Living status (co-residence) was divided into three groups: “with household member(s), “in an institution”, and “living alone.” Participants who habitually consumed alcohol, smoked cigarettes, or engaged in physical exercise were identified as “current drinkers,” “current smokers,” or “current exercisers,” respectively.

### 2.7 Statistical analysis

Continuous variables are presented as mean ± standard deviation (SD), while categorical variables are presented as frequencies and proportions (%). Data analyses were performed using SPSS software version 19 (SPSS Inc., Chicago, IL, USA) and AMOS version 23.0 (IBM, Armonk, NY, USA). Frequency analysis, reliability analysis, and correlational analysis were conducted using SPSS. Confirmatory factor analysis (CFA) and SEM were performed using AMOS. All reported *p*-values were based on two-sided tests.

## 3 Results

### 3.1 Characteristics of the study population

The final analytical sample included 9,859 older adults, with 45.9% males (*n* = 4,528) and 54.1% females (*n* = 5,331). The average age was 83.2 years (SD = 11.3), and the mean years of education was 3.7 years (SD = 4.5). Among the participants, 58.3% resided in urban areas, 16.0% were current smokers, 15.3% were current drinkers, and 35.0% reported engaging in regular physical exercise. In terms of living arrangements, 80.1% lived with household members, 16.4% lived alone, and 3.5% lived in institutions. Table 1 presents the detailed socio-demographic characteristics by sex.

**Table 1:** The characteristics of participants stratified by sex.

	All (*n* = 9,859)	Men (*n* = 4,528)	Women (*n* = 5,331)
Age (yrs)	83.2 ± 11.3	81.9 ± 10.6	84.4 ± 11.7
Education level (yrs)	3.7 ± 4.5	5.4 ± 4.6	2.4 ± 3.9
Residence, *n* (%)			
Urban	5,744 (58.3)	2,702 (59.7)	3,042 (57.1)
Rural	4,115 (41.7)	1,826 (40.3)	2,289 (42.9)
Current smokers, *n* (%)			
Yes	1,574 (16.0)	1,342 (29.6)	232 (4.4)
No	8,285 (84.0)	3,186 (70.4)	5,099 (95.6)
Current drinkers, *n* (%)			
Yes	1,507 (15.3)	1,186 (26.2)	321 (6.0)
No	8,352 (84.7)	3,342 (73.8)	5,010 (94.0)
Current exercisers, *n* (%)			
Yes	3,449 (35.0)	1,835 (40.5)	1,614 (30.3)
No	6,410 (65.0)	2,693 (59.5)	3,717 (69.7)
Living status, *n* (%)			
With household member(s)	7,900 (80.1)	3,786 (83.6)	4,114 (77.2)
Living alone	1,618 (16.4)	606 (13.4)	1,012 (19.0)
In an institution	341 (3.5)	136 (3.0)	205 (3.8)
Marital status, *n* (%)			
Married and living with spouse	4,644 (47.1)	2,841 (62.7)	1,803 (33.8)
Widowed	5,104 (51.8)	1,598 (35.3)	3,506 (65.8)
Others	111 (1.1)	89 (2.0)	22 (0.4)

Others: married but not living with spouse, divorced, or never married.

### 3.2 Confirmatory factor analysis (CFA)

Confirmatory factor analysis was conducted to assess the measurement model, including four latent variables: leisure activities, self-reported health, anxiety, and depression. All factor loadings were statistically significant, ranging from 0.57 to 0.92, indicating acceptable construct validity. Cronbach’s alpha coefficients ranged from 0.56 (leisure activities) to 0.92 (anxiety), suggesting acceptable to excellent reliability (54, 55) (Table 2).

**Table 2:** Confirmatory factor analysis: reliability, item loadings, and model fit indices.

Constructs	No. of item	Construct reliability (Cronbach’s α)	Indicator loadings
Leisure activities	5	0.56	0.57
SRH	2	0.57	0.62
Depression	10	0.81	0.83
Anxiety	7	0.92	0.92

Model fit indices indicated a good fit: RMSEA = 0.06, CFI = 0.91, NFI = 0.91, TLI = 0.90. All values exceeded the recommended thresholds (56–58), supporting the adequacy of the measurement model.

### 3.3 Correlations among variables

Pearson correlation analysis showed that leisure activities were positively associated with self-reported health (*r* = 0.20, *p* < 0.001), and negatively associated with depression (*r* = −0.13, *p* < 0.001) and anxiety (*r* = −0.05, *p* < 0.001). Self-reported health was negatively correlated with both depression (*r* = −0.26, *p* < 0.001) and anxiety (*r* = −0.19, *p* < 0.001), while anxiety was strongly and positively correlated with depression (*r* = 0.65, *p* < 0.001). All correlation coefficients were below 0.70, supporting discriminant validity (59) (Table 3).

**Table 3:** Correlations among constructs.

Constructs	1	2	3	4
1. Leisure activities	–			
2. SRH	0.20***	–		
3. Depression	−0.13***	−0.26***	–	
4. Anxiety	−0.05***	−0.19***	0.65***	–

***p < 0.001 for all correlations. All correlations are two-tailed.

### 3.4 SEM results for the structural model and hypotheses testing

The structural model demonstrated acceptable fit to the data: *p* < 0.001, RMSEA = 0.06, CFI = 0.91, TLI = 0.90.

All hypothesized direct paths were significant (Table 4). Specifically:

Leisure activities positively predicted self-reported health (*β* = 0.18, SE = 0.02, *t* = 11.58, *p* < 0.001), supporting H1.

Leisure activities negatively predicted depression (*β* = −0.16, SE = 0.01, *t* = −13.11, *p* < 0.001), supporting H2.

Leisure activities negatively predicted anxiety (*β* = −0.09, SE = 0.01, *t* = −6.20, *p* < 0.001), supporting H3.

Self-reported health negatively predicted depression (*β* = −0.33, SE = 0.01, *t* = −19.77, *p* < 0.001) and anxiety (*β* = −0.33, SE = 0.01, *t* = −19.49, *p* < 0.001), supporting H4 and H5.

Anxiety positively predicted depression (*β* = 0.49, SE = 0.02, *t* = 39.08, *p* < 0.001), supporting H6.

**Table 4:** Results of structural model testing.

Path	Path coefficient	S.E.	*t*	p
H1. Leisure activities → Self reported health	0.18	0.02	11.58	<0.001
H2. Leisure activities → Depression	−0.16	0.01	−13.11	<0.001
H3. Leisure activities → Anxiety	−0.09	0.01	−6.20	<0.001
H4. Self reported health → Depression	−0.33	0.01	−19.77	<0.001
H5. Self reported health → Anxiety	−0.33	0.01	−19.49	<0.001
H6. Anxiety → Depression	0.49	0.02	39.08	<0.001

*p < 0.001 for all correlations. All correlations are two-tailed.*

### 3.5 Indirect effects and mediation analysis

Indirect effects were tested using the bias-corrected bootstrap method with 5,000 resamples. The results revealed that self-reported health and anxiety significantly mediated the relationship between leisure activities and depression, supporting H7 (Table 5).

**Table 5:** Results of indirect effects of self-reported health and anxiety on depression.

Path	Indirect effect
H7-1. Leisure activities → Self reported health → Depression	−0.06
H7-2. Leisure activities → Anxiety → Depression	−0.04
H7-3. Leisure activities → Self reported health → Anxiety → Depression	−0.03

The indirect effect of leisure activities on depression via self-reported health was *β* = −0.06.

The indirect effect via anxiety was *β* = −0.04.

The serial mediation effect through both self-reported health and anxiety was *β* = −0.03.

## 4 Discussion

This study supports the proposed model that elucidates the interrelationships among leisure activities, SRH, anxiety, and depression among older adults in China. Specifically, leisure activities significantly enhanced SRH while reducing depression and anxiety. SRH contributed to the reduction of depression and anxiety, and anxiety had a direct positive impact on depression. Additionally, this study demonstrated that SRH and anxiety play partial and serial mediating roles in the relationship between leisure activities and depression.

The results for Hypothesis H1 revealed a positive relationship between leisure activities and SRH among older adults in China, extending previous literature related to older adults in other countries (15). H1 suggests that participants who reported higher levels of leisure activities were likely to have better SRH. As WHO and the International Society for Physical Activity and Health have pointed out, regular activity is essential for good mental health (60). Participation in leisure activities among older adults can effectively address their mental health issues (50) and thus improve SRH. Leisure activities can also indirectly enhance SRH by improving social support and interpersonal relationships (61).

The results for Hypotheses H2 and H3 demonstrated a negative relationship between leisure activities and depression/anxiety among older adults in China, consistent with previous studies (21). This finding can be explained by the fact that increased frequency of participation in leisure activities, such as playing cards/mahjong and watching/listening to TV/radio, is particularly important for reducing risk factors like social isolation among older adults and is associated with a lower risk of cognitive impairment (62) and lesser feelings of loneliness (63). Engaging in leisure activities helps older adults find hobbies and release psychological pressure, thereby reducing anxiety and depression (50).

In the study, we further confirmed that SRH was negatively associated with depressive and anxious symptoms among older adults in China (H4 and H5), which was consistent with previous analyses conducted in Western countries indicating that SRH was negatively associated with depression/anxiety among older adults (31). A study (64) participants reporting poor or fair self-rated health had greater odds of chronic illness, major depressive syndrome, and lower socioeconomic status than those reporting good to excellent self-rated health. Similarly, another study (65) revealed that SRH was a valid measure of physiological and mental health. These findings highlight the critical role of SRH as both an indicator and predictor of mental health outcomes, particularly in older populations.

The positive relationship between anxiety and depression (H6) among older adults in China was consistent with previous studies (66), indicating that coexisting anxiety and depression are common in the Chinese population. Epidemiological research has also shown that almost half of all people with depression also have comorbid anxiety (39).

Our findings empirically support the hypothesis that the indirect effect of leisure activities on depression among Chinese older adults can be mediated by SRH and anxiety (H7). Leisure activities can improve health, and older adults with rich leisure activities are more likely to report better health, which in turn alleviates their anxiety and depression. The findings of this study enrich our understanding of the relationship between leisure activities and depression and provide a basis for policy interventions. On one hand, SRH is a predictor of anxiety and depression, as indicated by previous studies (29). When SRH worsens, it is crucial to intervene to prevent anxiety and depression. On the other hand, anxiety serves as a mediator not only between leisure activities and depression but also between SRH and depression. Anxiety has been found to be a burdensome mental health issue and is often accompanied by emotional irritation, frequent anger, sleep disorders, muscle pain, and other symptoms (67). Among older adults, anxiety can lead to cognitive impairment (68), a decline in subjective well-being (69), and lower life satisfaction (70). Policy interventions targeting depression should place greater emphasis on alleviating anxiety.

## 5 Strengths and limitations

This study has several notable strengths. Firstly, it utilizes a large and representative sample from China, enhancing the generalizability of the findings and providing valuable references for similar research in other countries. Secondly, the study not only explores the direct relationship between leisure activities and depression among older adults but also analyzes the mediating roles of SRH and anxiety. This multi-faceted approach enriches our understanding of the complex interactions among these variables. Additionally, the CES-D-10 and GAD-7 scales used in this study have been validated among Chinese older adults, ensuring the reliability and validity of the measurement tools.

However, there are some limitations to this study. Firstly, the assessment of depression and anxiety symptoms relied on the CES-D-10 and GAD-7 scales rather than clinical diagnostic criteria. Although these scales are validated, they may not fully capture professional clinical diagnoses. Secondly, the use of self-report questionnaires may introduce recall bias and subjective perception issues, potentially affecting the accuracy of the data. Lastly, the cross-sectional design of this study limits the ability to infer causality between the variables. Longitudinal studies are needed in the future to establish causal relationships and examine the temporal dynamics of these associations.

## 6 Conclusion

This study, based on data from the CLHLS, used structural equation modeling to reveal the relationships among leisure activities, SRH, anxiety, and depression in older adults in China. The findings indicate that leisure activities not only directly improve SRH and alleviate anxiety and depression but also exert indirect and serial mediation effects through SRH and anxiety. SRH emerged as a key indicator of mental health, playing an important role in preventing anxiety and depression, while anxiety significantly increased the risk of depression.

Theoretically, this study expands the research framework on mental health in older adults by confirming that the positive effects of leisure activities also apply in the Chinese context. It is the first to clearly identify the mediating roles of SRH and anxiety in these relationships. Practically, the findings provide valuable recommendations for governments, communities, and healthcare institutions: promoting leisure activities such as gardening, reading, and social engagement can effectively enhance subjective health and psychological well-being among older adults, reducing the incidence of depression and anxiety. Support and encouragement from family members and caregivers also play a critical role in facilitating participation.

Overall, this study highlights the importance of leisure activities as a strategic approach to promoting mental health in older adults. It calls for collaborative efforts across sectors to address the mental health challenges posed by China’s rapidly aging population, offering both scientific evidence and practical solutions.

## Data Availability

The original contributions presented in the study are included in the article/supplementary material, further inquiries can be directed to the corresponding author/s.
